# Assessment of Self-Perceived Hearing Disability Using the Arabic Speech, Spatial, and Qualities of Hearing Scale-12 (SSQ-12) Among Adults Referred for Contralateral Routing of Signal/Bilateral Contralateral Routing of Signal (CROS/BiCROS) Evaluation

**DOI:** 10.7759/cureus.114536

**Published:** 2026-08-14

**Authors:** Raghad A Alrumaih, Mashael A Alshayie, Abdulrahim H Alshehri, Lara I Binbrikan, Faisal H Alotaibi, Nora M Almegil

**Affiliations:** 1 Department of Audiology, General Directorate of Medical Services, Ministry of Interior, Saudi Arabia, Security Forces Hospital Program, Riyadh, SAU

**Keywords:** arabic, bicros, cros, psychometrics, self-perceived hearing disability, single-sided deafness, unilateral hearing loss

## Abstract

Background: Unilateral hearing loss (UHL), single-sided deafness (SSD), and asymmetric hearing loss (AHL) impair speech understanding in noise, sound localization, and overall communication. These conditions are commonly managed with contralateral routing of signal (CROS) or bilateral CROS (BiCROS) hearing aids. Culturally appropriate, psychometrically sound patient-reported outcome measures are needed to characterize self-perceived hearing disability in Arabic-speaking populations prior to fitting.

Objective: To characterize self-perceived hearing disability and evaluate the psychometric properties (internal consistency, floor/ceiling effects, and subscale intercorrelations) of the Arabic Speech, Spatial, and Qualities of Hearing Scale, 12-item version (SSQ-12), among adults referred for CROS/BiCROS evaluation.

Methods: In this cross-sectional observational study, 54 adults referred for CROS/BiCROS evaluation at the Audiology Department, Security Forces Hospital Program, General Directorate of Medical Services, Ministry of Interior, Riyadh, Saudi Arabia, were recruited via consecutive convenience sampling. Eligible participants were aged ≥18 years, had confirmed UHL, SSD, or AHL, and had no cognitive, psychiatric, or communication disorders precluding reliable self-report. Participants completed the Arabic SSQ-12, a 12-item patient-reported outcome measure rated on an 11-point scale from 1 (poorest) to 10 (best), with a not applicable (N/A) option, covering speech perception (items 1-5), spatial hearing (items 6-9), and sound quality (items 10-12). The questionnaire was completed independently or with the assistance of an audiologist before hearing aid fitting. Descriptive statistics (frequencies/percentages, mean ± SD), item- and subscale-level floor/ceiling effects (>15% scoring 1 or 10), internal consistency (Cronbach’s α), and subscale intercorrelations (Pearson’s r) were computed.

Results: The total SSQ-12 score was 3.82 ± 2.17 (scale 1-10), with subscale means ranging from 3.54 ± 2.28 (speech perception) to 4.43 ± 2.64 (sound quality); all fell below the scale midpoint. Item SSQ6 (locating sound source direction) had the lowest mean (3.15), and 11 of 12 items showed floor effects exceeding 15%. The total SSQ-12 demonstrated excellent internal consistency (α = 0.944), and the speech perception (α = 0.885) and spatial hearing (α = 0.901) subscales showed good-to-excellent reliability, whereas the three-item sound quality subscale showed lower reliability (α = 0.548). Speech perception and spatial hearing were strongly correlated (r = 0.756, p < 0.001).

Conclusion: Adults referred for CROS/BiCROS evaluation report substantial self-perceived hearing disability across speech, spatial, and sound-quality domains. The Arabic SSQ-12 total scale shows excellent internal consistency and captures this disability profile, supporting its use as a routine outcome measure in Arabic-speaking CROS/BiCROS candidates, while the brief sound quality subscale warrants cautious interpretation.

## Introduction

Unilateral hearing loss (UHL), encompassing single-sided deafness (SSD) and asymmetric hearing loss (AHL), describes a substantial hearing deficit in one ear with better hearing in the contralateral ear. Loss of binaural input impairs sound localization and speech understanding in noise, and profound unilateral loss is associated with reduced socialization, learning difficulty, and diminished work productivity [1]. UHL is common: the US national survey data report a prevalence of approximately 8% among adults, with subjective communication difficulty roughly three times more frequent than among normal-hearing peers [2], and asymmetric hearing is similarly prevalent, particularly with advancing age [3].

Saudi Arabia's national disability survey data indicate that hearing impairment affects a meaningful share of the population, with regional variation in prevalence and service access [4], and clinical series from Saudi ear, nose, and throat (ENT) centers suggest hearing loss may be more prevalent than previously estimated [5,6]. The contributing factors include a comparatively high rate of consanguineous marriage, raising the risk of hereditary hearing loss [7]; under-recognized occupational noise exposure, particularly among military personnel [8,9]; and gaps in noise-related awareness [10]. Screening pathways also appear inconsistent, with low uptake of adult hearing screening [11] and variable parental awareness of newborn screening outcomes [12], suggesting many UHL, SSD, and AHL cases may present later than optimal, reinforcing the value of a validated, easily administered self-report tool once patients reach specialist care.

Contralateral routing of signal (CROS) and bilateral CROS (BiCROS) hearing aids are among the most widely used non-surgical rehabilitation options for UHL, SSD, and AHL, transmitting sound from the impaired ear to the better-hearing ear. Recent longitudinal work suggests CROS/BiCROS use can improve speech-in-noise performance and reduce listening effort [13], yet these devices do not restore true binaural hearing, and self-perceived disability commonly persists even with successful fitting; randomized trial evidence comparing CROS and bone conduction devices with cochlear implantation for SSD confirms that non-surgical options do not fully normalize binaural function, though many patients derive subjective benefit [14,15]. Because audiometric measures do not fully capture the functional impact of hearing loss, patient-reported outcome measures (PROMs) are an essential complement to the audiogram when evaluating and monitoring CROS/BiCROS candidates.

The Speech, Spatial, and Qualities of Hearing Scale (SSQ) and its 12-item form (SSQ-12) are among the most widely used PROMs for this purpose, assessing speech perception, spatial hearing, and qualities of hearing [16,17]. The SSQ-12 has been cross-culturally validated in numerous languages, including Spanish [18] and Romanian [19], and an Arabic version was validated by Alkhodair et al. [20] in a general hearing-impaired population. However, data on the Arabic SSQ-12 specifically among patients evaluated for CROS/BiCROS fitting, a population whose disability profile is driven by loss of binaural cues rather than bilateral symmetric loss, remain limited.

The present study therefore had two aims: first, to characterize the self-perceived hearing disability profile, at both the subscale and item level, of adults referred for CROS/BiCROS evaluation using the Arabic SSQ-12; and second, to evaluate the psychometric performance of the Arabic SSQ-12 in this population, including its internal consistency, floor and ceiling effects, and subscale intercorrelations. These findings are intended to inform the clinical use of the Arabic SSQ-12 as a routine pre-fitting assessment tool and to identify domains of hearing disability most relevant to counseling and rehabilitation planning.

## Materials and methods

Study design

This cross-sectional observational study characterized self-perceived hearing disability and evaluated the psychometric properties of the Arabic version of the SSQ-12 [20] in adults referred for CROS or BiCROS evaluation.

Study setting

The study was conducted in the Audiology Department, Security Forces Hospital Program, General Directorate of Medical Services, Ministry of Interior, Saudi Arabia, over a four-month period from February 15 to July 9, 2026. Participants were recruited through routine referrals to the Audiology Department for CROS/BiCROS evaluation. All questionnaire administration, data collection, and statistical analyses were conducted within the Audiology Department.

Study population

The study population comprised adults referred to the audiology department for CROS or BiCROS evaluation.

Inclusion criteria

Patients were eligible if they were aged 18 years or older, had a confirmed diagnosis of UHL, SSD, or AHL, were referred for CROS/BiCROS evaluation, and had completed the Arabic SSQ-12 prior to fitting.

Exclusion criteria

Patients were excluded if they were younger than 18 years, had symmetric hearing loss, had diagnosed cognitive, psychiatric, or communication disorders that could compromise reliable self-report, had duplicate records, were identified as non-patient or test entries, or did not meet the predefined inclusion criteria.

Sampling technique

A convenience sampling strategy was employed. All eligible patients presenting to the Audiology Department during the study period were approached for inclusion, yielding a consecutive clinical sample. No formal a priori sample size calculation was performed because this was a cross- sectional observational study using a consecutive convenience sample. All eligible adults referred for CROS/BiCROS evaluation during the study period who met the inclusion criteria were invited to participate, and all consenting participants were included in the study.

Data collection tool

Self-perceived hearing ability was assessed using the Arabic SSQ-12, a validated 12-item PROM derived from the original 49-item Speech, Spatial, and Qualities of Hearing Scale (SSQ). The SSQ-12 comprises three subscales: speech perception (items 1-5), assessing speech understanding in varied listening environments; spatial hearing (items 6-9), assessing perception of sound direction and distance; and sound qualities (items 10-12), assessing identification, segregation, and naturalness of sounds. Each item is rated on an 11-point scale from 1 (poorest) to 10 (best) self-perceived ability, with a not applicable (N/A) option for items considered irrelevant; N/A responses are excluded from score computation for that item. Subscale scores are the mean of non-N/A items, and the total SSQ-12 score is the mean of all scored items, with higher scores indicating better function. The questionnaire was administered for research purposes only, after participants had already been identified as CROS/BiCROS candidates based on clinical audiological assessment, and was not used to determine clinical candidacy or hearing aid selection.

Patients referred to the Audiology Department for CROS/BiCROS evaluation underwent routine audiological assessment (pure-tone audiometry) to confirm UHL/SSD/AHL. Eligible patients were then invited to participate, informed consent was obtained, and the Arabic SSQ-12 was self-administered (or administered by a trained audiologist for patients requiring assistance) in a quiet clinic room prior to CROS/BiCROS fitting. Completed questionnaires were checked for completeness at the point of collection, anonymized, and entered into Microsoft Excel 2021 (Microsoft Corporation, Redmond, WA) for statistical analysis.

Variables

Demographic and clinical variables included age category (19-30, 31-40, 41-50, 51+ years), gender, affected ear (right, left, or bilateral asymmetric), duration of hearing loss, and prior hearing aid history. Outcome variables comprised individual item scores (SSQ1-12) and the speech perception (items 1-5), spatial hearing (items 6-9), sound quality (items 10-12), and total SSQ-12 subscale/total scores.

Data analysis

All statistical analyses were performed using Microsoft Excel 2021. Descriptive statistics were computed for all demographic and clinical variables, with categorical variables reported as frequencies and percentages and continuous variables as mean ± SD, median, and range. SSQ-12 profile analysis computed mean ± SD for the speech perception, spatial hearing, sound quality, and total SSQ-12 scores. Item-level analysis identified the items with the lowest and highest mean scores to reveal specific domains of maximal disability. Floor and ceiling effects were evaluated for each item, defined as the percentage of respondents scoring 1 (floor) or 10 (ceiling); effects exceeding 15% were considered clinically significant, consistent with thresholds used in the broader quality-of-life measurement literature [21]. Internal consistency was assessed using Cronbach's alpha for the total scale and each subscale, with α ≥ 0.70 considered acceptable, ≥ 0.80 good, and ≥ 0.90 excellent. Subscale intercorrelations were quantified using Pearson's r; because two participants lacked a subscale score due to all-N/A responses, the effective sample for three-subscale analyses was 52. Two-tailed tests were applied (p < 0.05), with correlations interpreted as weak (r < 0.30), moderate (0.30-0.60), or strong (≥ 0.60). All questionnaire data and statistical outputs were reviewed and interpreted by senior audiologists.

Ethical approval

Ethical approval for this study was obtained from the Institutional Review Board (IRB), Security Forces Hospital Program, General Directorate of Medical Services, Ministry of Interior, Saudi Arabia (Research No. 26-918-48; IRB Registration No. H-01-R-069). The study was conducted in accordance with the ethical principles of the Declaration of Helsinki. Informed consent was obtained from all participants before completing the Arabic SSQ-12 questionnaire. All collected data were anonymized prior to analysis, and no personally identifiable information was retained.

## Results

Study sample

A total of 54 participants were enrolled and included in the final analysis. Item-level response counts varied because the questionnaire permits participants to select not applicable (N/A) for irrelevant items; N/A is a valid response option, not missing data, and does not result in exclusion. A subscale score is calculable provided at least one item within that subscale received a numeric response. Two participants selected N/A for an entire subscale: one for all five speech perception items and one for all three sound quality items. Consequently, the effective sample size was 53 for speech perception and sound quality and 54 for spatial hearing; both participants are included in analyses for which they have a valid score.

Demographic and clinical characteristics

The demographic and clinical characteristics of the participants are presented in Table 1.

**Table 1:** Demographic and clinical characteristics of the participants (N = 54).

Characteristic	n (%)
Age category (years)	
19-30	5 (9.3)
31-40	4 (7.4)
41-50	14 (25.9)
51+	31 (57.4)
Gender	
Female	30 (55.6)
Male	24 (44.4)
Affected ear	
Right	24 (44.4)
Left	23 (42.6)
Bilateral asymmetric	7 (13.0)
Prior hearing aid history	
No previous hearing aid use	48 (88.9)
Previous hearing aid user	6 (11.1)

The study enrolled 54 participants, including 30 females (55.6%) and 24 males (44.4%), most aged 51 years or older (n = 31; 57.4%), followed by 41-50 years (n = 14; 25.9%), 19-30 years (n = 5; 9.3%), and 31-40 years (n = 4; 7.4%). The right ear was most commonly affected (n = 24; 44.4%), followed by the left (n = 23; 42.6%), with bilateral asymmetric involvement in seven participants (13.0%). Most participants had no prior hearing aid experience (n = 48; 88.9%); only six (11.1%) had used one previously. The mean duration of hearing loss was 21.5 ± 17.7 years (range: 2-70), reflecting a heterogeneous sample.

SSQ-12 profile

The total and subscale SSQ-12 scores are summarized in Table 2.

**Table 2:** SSQ-12 total and subscale scores. SSQ-12: Speech, Spatial, and Qualities of Hearing Scale, 12-item version.

Scale/Subscale	N	Mean ± SD
Speech perception (Items 1-5)	53	3.54 ± 2.28
Spatial hearing (Items 6-9)	54	3.58 ± 2.57
Sound quality (Items 10-12)	53	4.43 ± 2.64
Total SSQ-12 score	54	3.82 ± 2.17

The mean subscale scores ranged from 3.54 ± 2.28 (speech perception) to 4.43 ± 2.64 (sound quality); the total SSQ-12 score was 3.82 ± 2.17. Speech perception and spatial hearing yielded the lowest scores, while sound quality yielded the highest; all scores fell below the scale midpoint of 5.5.

Item-level analysis

The item-level SSQ-12 scores for the 54 participants are presented in Table 3.

**Table 3:** Item-level SSQ-12 scores (N = 54). SSQ-12: Speech, Spatial, and Qualities of Hearing Scale, 12-item version.

Item	Description/Domain	Mean ± SD
SSQ1	Speech - Following conversation with one other person (Speech perception)	3.57 ± 3.08
SSQ2	Speech - Following conversation with background noise (Speech perception)	3.62 ± 3.21
SSQ3	Speech - Following rapid speech (Speech perception)	3.57 ± 2.63
SSQ4	Speech - Telephone conversation (Speech perception)	3.69 ± 2.85
SSQ5	Speech - Following group conversations (Speech perception)	3.21 ± 2.43
SSQ6	Spatial - Locating sound source direction (Spatial hearing)	3.15 ± 2.75
SSQ7	Spatial - Detecting whether a sound is moving (Spatial hearing)	3.48 ± 2.91
SSQ8	Spatial - Judging distance of sound source (Spatial hearing)	3.59 ± 2.78
SSQ9	Spatial - Following direction of sounds in a dynamic environment (Spatial hearing)	3.86 ± 3.01
SSQ10	Quality - Ease of distinguishing different sound types (Sound quality)	3.33 ± 2.90
SSQ11	Quality - Naturalness of own voice quality (Sound quality)	4.60 ± 3.40
SSQ12	Quality - Clarity and richness of environmental sounds (Sound quality)	4.80 ± 3.16

The mean item scores ranged from 3.15 (SSQ6: locating sound direction) to 4.80 (SSQ12: clarity/richness of environmental sounds). The lowest-scoring items were SSQ6, SSQ5 (3.21), and SSQ10 (3.33); the highest were SSQ12, SSQ11 (4.60), and SSQ9 (3.86). All item means fell below 5.0 on the 1-10 scale.

Floor and ceiling effects

The floor and ceiling effects for each SSQ-12 item are presented in Table 4.

**Table 4:** Floor and ceiling effects for each SSQ-12 item (N = 54). Note: Values marked with an asterisk (*) indicate floor or ceiling effects exceeding the conventional 15% criterion for identifying floor or ceiling effects. SSQ-12: Speech, Spatial, and Qualities of Hearing Scale, 12-item version.

Item	Domain	Floor effect (Score = 1), n (%)	Ceiling effect (Score = 10), n (%)
SSQ1	Speech perception	11 (20.4%)	4 (7.4%)
SSQ2	Speech perception	23 (42.6%)*	8 (14.8%)
SSQ3	Speech perception	17 (31.5%)*	4 (7.4%)
SSQ4	Speech perception	18 (33.3%)*	4 (7.4%)
SSQ5	Speech perception	16 (29.6%)*	3 (5.6%)
SSQ6	Spatial hearing	22 (40.7%)*	4 (7.4%)
SSQ7	Spatial hearing	19 (35.2%)*	5 (9.3%)
SSQ8	Spatial hearing	16 (29.6%)*	3 (5.6%)
SSQ9	Spatial hearing	14 (25.9%)*	4 (7.4%)
SSQ10	Sound quality	21 (38.9%)*	4 (7.4%)
SSQ11	Sound quality	11 (20.4%)*	11 (20.4%)*
SSQ12	Sound quality	8 (14.8%)	10 (18.5%)*

Floor effects (score = 1) exceeded the 15% threshold for 11 of 12 items, highest for SSQ2 (42.6%), SSQ6 (40.7%), and SSQ10 (38.9%) (see Table 4 for all values); only SSQ12 (14.8%) did not exceed this threshold. Ceiling effects remained below 15% for most items except SSQ11 (20.4%) and SSQ12 (18.5%). Percentages are relative to all scored (non-N/A) responses per item.

Reliability analysis

The internal consistency reliability results for the Arabic SSQ-12 are shown in Table 5.

**Table 5:** Internal consistency reliability of the Arabic SSQ-12 (N = 54). SSQ-12: Speech, Spatial, and Qualities of Hearing Scale, 12-item version.

Scale/Subscale	No. of Items	Cronbach's alpha (α)
Total SSQ-12	12	0.944
Speech perception	5	0.885
Spatial hearing	4	0.901
Sound quality	3	0.548

The total Arabic SSQ-12 demonstrated excellent internal consistency (α = 0.944); the speech perception (α = 0.885) and spatial hearing (α = 0.901) subscales showed good-to-excellent reliability, while sound quality was lower (α = 0.548).

Subscale intercorrelations

**Figure 1:**
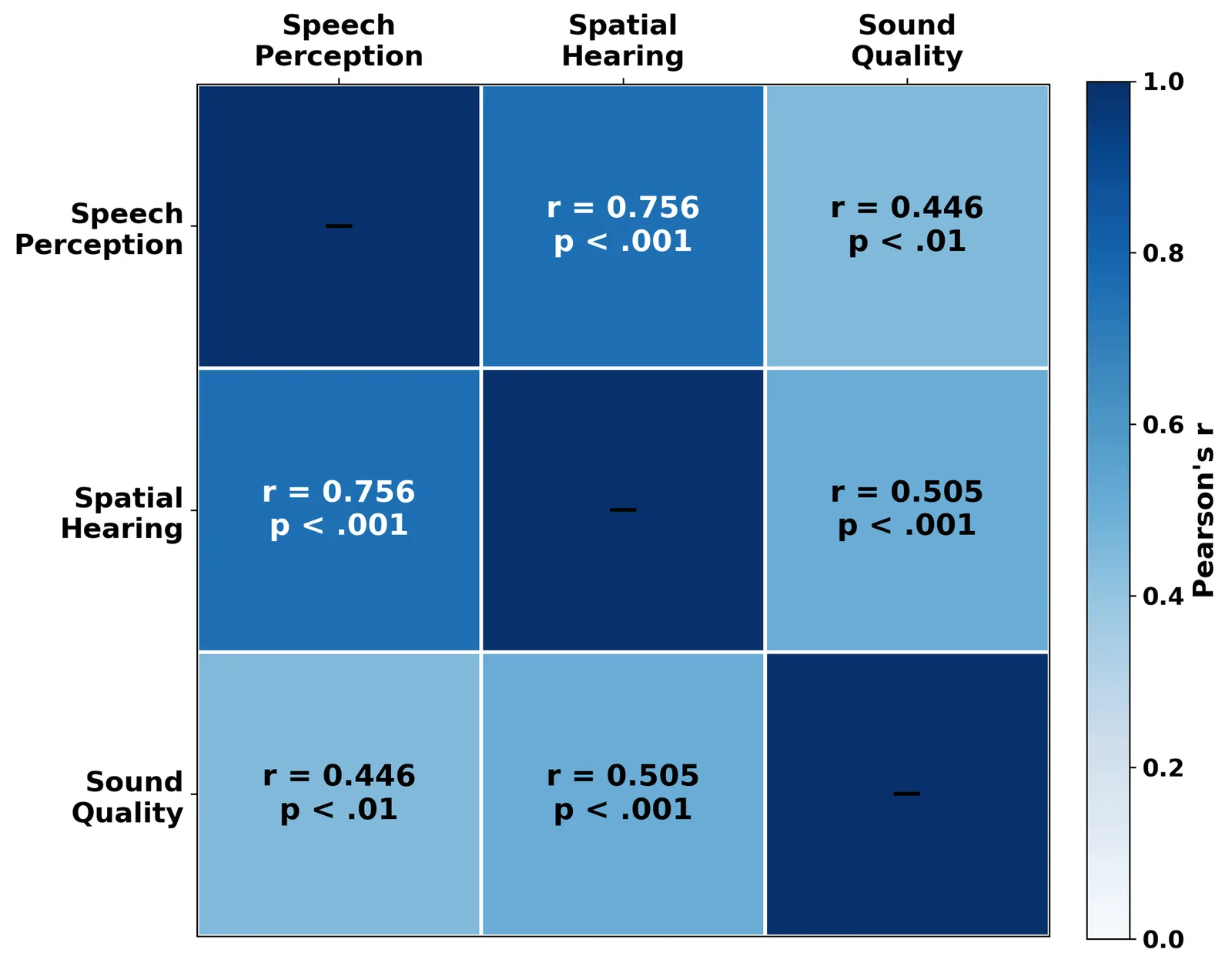
Pearson correlation between SSQ-12 subscales (n = 52). SSQ-12: Speech, Spatial, and Qualities of Hearing Scale, 12-item version.

The Pearson correlations among the three SSQ-12 subscales are presented in Figure 1.

All three pairwise intercorrelations were positive and significant: strong between speech perception and spatial hearing (r = 0.756, p < 0.001), and moderate between spatial hearing and sound quality (r = 0.505, p < 0.001) and speech perception and sound quality (r = 0.446, p < 0.01).

## Discussion

In this sample of 54 adults referred for CROS/BiCROS evaluation, self-perceived hearing disability was substantial and pervasive: all subscale and total scores fell well below the scale midpoint, and 11 of 12 items showed floor effects exceeding 15%. This pattern fits the pathophysiology of unilateral and asymmetric hearing loss, in which loss of binaural input, rather than a global reduction in loudness perception, drives much of the reported difficulty, since interaural timing and level cues normally allow the auditory system to segregate and localize competing sound sources [1]. The item-level findings support this mechanism: the lowest-scoring item overall was SSQ6 (locating sound direction), a task depending almost entirely on binaural comparison. Spatial hearing and speech perception were similarly the two lowest-scoring domains and were strongly intercorrelated (r = 0.756, p < 0.001), suggesting they capture closely related aspects of a single binaural-hearing construct. Sound quality, addressing sound segregation, identification, and naturalness (including one's own voice), scored higher and correlated only moderately with the other two subscales, consistent with a partially distinct dimension of listening experience.

The excellent internal consistency of the total SSQ-12 (α = 0.944) and good-to-excellent reliability of the speech perception (α = 0.885) and spatial hearing (α = 0.901) subscales are consistent with the original Arabic SSQ-12 validation [20] and with strong estimates for other adaptations, including the Spanish [18] and Romanian [19] versions, supporting the total scale's reliability as a robust, generalizable property rather than a sample-specific artifact. The lower alpha for the three-item sound quality subscale (0.548) warrants more cautious interpretation, as it spans conceptually varied aspects with only three items and is more susceptible to attenuated reliability. This is reinforced by the floor and ceiling effect analysis: effects at or above 15% are considered to compromise a scale's ability to discriminate between patients and detect change over time [21], and the near-universal floor effects observed here (11 of 12 items) indicate limited sensitivity to detect further deterioration at the low end in this population. The ceiling effects for SSQ11 and SSQ12 (both sound quality items) suggest that, for a subset of participants, sound quality remains relatively well preserved despite pronounced speech and spatial difficulty, reinforcing a domain-specific rather than uniform disability profile.

These findings should be considered against current CROS/BiCROS outcomes and the broader SSD/AHL treatment landscape. Longitudinal evidence indicates CROS/BiCROS use can meaningfully improve speech-in-noise performance and reduce listening effort over time [13], and because CROS/BiCROS reroutes rather than restores binaural input, the pronounced pre-fitting disability observed here provides a meaningful baseline for future rehabilitation comparisons. CROS/BiCROS fitting is also only one of several SSD treatment pathways; patient motivations strongly influence whether patients pursue amplification, a bone conduction device, or cochlear implantation [22], suggesting these findings could usefully inform counseling about the relative merits of each option. The sample's demographic profile, dominated by adults aged 51 years and older, is consistent with age-related causes of asymmetric hearing loss described in Saudi [4] and broader [3] epidemiological data.

Limitations

Several limitations should be considered. The cross-sectional, single-center design, using a convenience sample from one Riyadh audiology department, limits generalizability. The study lacked a normal-hearing or bilaterally symmetric comparison group, so discriminant validity could not be assessed, and test-retest reliability and responsiveness to change were not evaluated, as participants completed the SSQ-12 only once, before fitting. Audiometric findings were not correlated with SSQ-12 scores, precluding assessment of how closely self-perceived disability tracks objective hearing status. The modest sample size, and smaller subscale-level samples after N/A exclusions, particularly limited the precision of the three-item sound quality subscale's reliability estimate. As with any self-report instrument, responses may be influenced by recall bias, social desirability, or response style, without independent corroboration. Finally, because enrollment relied on patients already referred for CROS/BiCROS evaluation, the sample may not capture individuals with UHL, SSD, or AHL who did not seek or receive referral, potentially limiting broader applicability.

## Conclusions

Adults referred for CROS/BiCROS evaluation in this Saudi tertiary-care sample reported considerable self-perceived hearing disability across all three SSQ-12 domains, with the greatest difficulty in speech perception and spatial hearing, a pattern consistent with the loss of binaural hearing cues that characterizes unilateral and asymmetric hearing loss. The Arabic SSQ-12 total scale demonstrated excellent internal consistency in this population, supporting its use as a practical, brief outcome measure for characterizing hearing-related disability in Arabic-speaking CROS/BiCROS candidates. The lower reliability of the three-item sound quality subscale and the extensive floor effects observed across most items indicate that subscale-level and low-end scores should be interpreted cautiously. Future research should prioritize multi-center recruitment, inclusion of comparison groups, correlation of SSQ-12 scores with audiometric findings, and longitudinal pre- and post-fitting assessment to establish the responsiveness of the Arabic SSQ-12 and to more directly quantify the functional benefit of CROS/BiCROS rehabilitation in this population.
